# Texture Properties and Chewing of κ-Carrageenan–Konjac Gum–Milk Hydrogels Modified with Carrot Callus Cells

**DOI:** 10.3390/gels11120990

**Published:** 2025-12-09

**Authors:** Elena Günter, Oxana Popeyko, Natalia Zueva, Inga Velskaya, Fedor Vityazev, Sergey Popov

**Affiliations:** Institute of Physiology of Federal Research Centre “Komi Science Centre of the Urals Branch of the Russian Academy of Sciences”, 50, Pervomaiskaya Str., 167982 Syktyvkar, Russia; gunter-ea@mail.ru (E.G.); opopeyko@mail.ru (O.P.); nata.zueva.2000@mail.ru (N.Z.); velskaya.psy@gmail.com (I.V.); rodefex@mail.ru (F.V.)

**Keywords:** carrot callus, mechanical properties, texture, sensory properties, chewing, κ-carrageenan–konjac gum hydrogels

## Abstract

This study aims to assess the effect of carrot callus cells on the mechanical and textural qualities, including chewing parameters, of κ-carrageenan–konjac gum–milk hydrogels. The mechanical and textural qualities were assessed instrumentally with a texture analyzer and using sensory analysis with untrained volunteers (*n* = 31), respectively. The mechanical properties of both cell-free and cell-encapsulated hydrogels were found to increase with an increase in gel concentration from 0.4 to 1.0%. The instrumentally measured hardness increased by 7–10% in 0.4% and 1.0% gels at 20 and 60% cell concentrations, respectively. The springiness, cohesiveness, and gumminess of the hydrogels decreased with an increase in the cell concentration. The overall liking did not change with the addition of cells, except for the liking scores of the 0.4% hydrogel containing 60% cells, which decreased. Adding 60% cells to the 0.4% hydrogel improved perceived hardness and adhesiveness. The graininess ratings were positively correlated with the cell concentration and negatively correlated with elasticity and cohesiveness, but were not associated with the instrumental hardness and gumminess. The change in sensory assessments resulting from the addition of cells was accompanied by increased masticatory muscle activity during hydrogel chewing. Thus, the incorporation of plant cells into gum hydrogel represents a promising approach to creating unique gel textures and developing innovative functional foods.

## 1. Introduction

The use of polysaccharide hydrogels is popular in the food sector for developing products with improved mechanical and sensory properties [1,2]. Plant cell and tissue cultures have recently been used to modify the mechanical characteristics of food hydrogels through methods such as the 3D bioprinting approach [3,4,5,6,7,8,9,10,11,12,13,14]. Plant cells give hydrogels a unique texture similar to artificial plant tissues [4,5,6], which is promising for creating healthy functional foods with a specific food texture.

It has been shown that the effect of incorporating plant cells into hydrogels depends on both the hydrogel composition and the type of cells used. Several studies have shown that the inclusion of plant cells reduces the mechanical properties of hydrogels. Specifically, the addition of carrot callus cells to an alginate gel [6], duckweed and campion callus cells to an alginate hydrogel [15], lettuce leaf cells [5] to a low-methylesterified pectin hydrogel, and duckweed callus cells to a κ-carrageenan hydrogel [13] reduced the mechanical properties of the hydrogels. Contrarily, incorporating duckweed callus cells into weak hydrogels based on citrus pectin contributed to an increase in hydrogel hardness [13]. Increasing hardness is a promising approach in the design of food gels with improved food quality. Increasing food hardness can provide increased hedonic scores [16,17,18], slower eating rates, and increased satiety [19], and dietary hardness has a beneficial impact on behavior, cognition, and brain function [20].

In a previous study, we showed that adding carrot callus cells to model weak xanthan–konjac gels enhanced the mechanical and rheological properties of the final gel product, as well as increased the perception of graininess by humans [12]. Carrot cells are characterized by a high content of carotenoids and possess antioxidant properties [21], making them an important ingredient in food gels with high nutritional value. It is logical to assume that the higher the content of carrot cells in a gel product, the greater its benefits may be. However, the low hardness of xanthan–konjac gels (ca. 1 N) and the low content of carrot cells in it (20%) required further improvement of the gel product, which was developed.

In the present study, the κ-carrageenan–konjac gum–milk hydrogel system was used to obtain a gel with the desired properties. Hydrogels based on κ-carrageenan and konjac gum are used in the food industry as gelling agents, thickeners, and stabilizers [22,23]. Monosulfated κ-carrageenan is extracted from red seaweed and consists of an alternating linear chain of α(1→3)-D-galactose-4-sulfate and β(1→4)-3,6-anhydro-D-galactose [24]. Konjac gum is obtained from the tubers of the Amorphophallus konjac plant and is a glucomannan consisting of a linear chain of 1,4-linked D-mannose and D-glucose residues in a ratio of 1.6:1 [25,26]. Acetyl groups are randomly present at the C-6 position along the glucomannan [25,26]. Konjac gum forms gels with weak mechanical properties, so it is often used in a mixture with other polysaccharides such as κ-carrageenan and xanthan gum [23,26]. Low concentrations of these polysaccharides can alter the textural and sensory characteristics of food [22,23,26]. In addition to polysaccharides, proteins are often used as an ingredient in food systems. Casein is the main protein component of cow’s milk and is widely used in food production due to its properties such as foaming, water binding, and thickening [22]. Incorporating proteins and polysaccharides into a single product can induce intermolecular interactions, forming a mixed network of κ-carrageenan and casein, and improve its functional properties [22]. It has been shown that the inclusion of konjac gum synergistically improved the gelling properties of whey protein and contributed to the formation of a denser micro-network structure [27]. The addition of konjac gum significantly improved the texture of the mixed gel based on salmon myofibrillar protein and konjac gum, which exhibited more resilient and stable properties in the oral cavity [28].

For further development of this field, first of all, it is crucial to investigate the effect of plant cell encapsulation on the mechanical properties of hydrogels. Mechanical properties are known to have a significant impact on hedonic appraisal and food choice [29]. For example, the “graininess” feature had a significant impact on cheese liking scores [30]. A grainy perception of yogurt has been found to be favorably correlated with particle size but negatively correlated with overall liking, taste, and flavor liking [31]. However, the effect of encapsulating plant cells in hydrogels on their mechanical properties and human texture perception, specifically the graininess, is not fully understood. For instance, previously Park et al. [6] incorporated live carrot callus cells into a hydrogel system based on a 4% alginate gel and only investigated the effect of adding carrot cells on gel hardness. However, the cells’ impact on other mechanical and rheological properties or human texture perception was not examined. A novel aspect of this study is that to create food gels with desired properties (mechanical, rheological, and functional), we modified the mechanical properties of hydrogels based on κ-carrageenan, konjac gum, and milk by enriching them with carrot callus culture cells. We hypothesize that encapsulating callus cells will improve the mechanical (hardness, cohesiveness, gumminess, and springiness), rheological, and functional (human texture perception) properties of food hydrogels. The use of polysaccharide/milk gels will allow for the creation of mixed gels with a wide range of hardness and variable rheology due to the formation of a denser structure in such gels. We applied an integrative approach, which included creating plant cell-loaded hydrogel models and subsequently studying their mechanical, rheological, textural, chewing, and sensory properties in relation to each other. The obtained data will contribute to clarifying the influence of plant cells on the mechanical and rheological properties of food gels, as well as on human perception of their texture, which is important for the development of healthy functional products with improved mechanical, textural, and sensory properties.

The aim of the study was to determine the effect of adding carrot callus cells on the mechanical, rheological, and textural properties of κ-carrageenan–konjac gum–milk hydrogels. The chewing characteristics of plant cell-enriched hydrogels were also studied, as chewing influences sensory perception. Moreover, the influence of callus cells on sensory and chewing characteristics was separately assessed in subgroups of volunteers with normal and increased body weight, since food texture has been demonstrated to have a substantial influence on body weight.

## 2. Results and Discussion

### 2.1. Microstructure of Hydrogels

**Figure 1:**
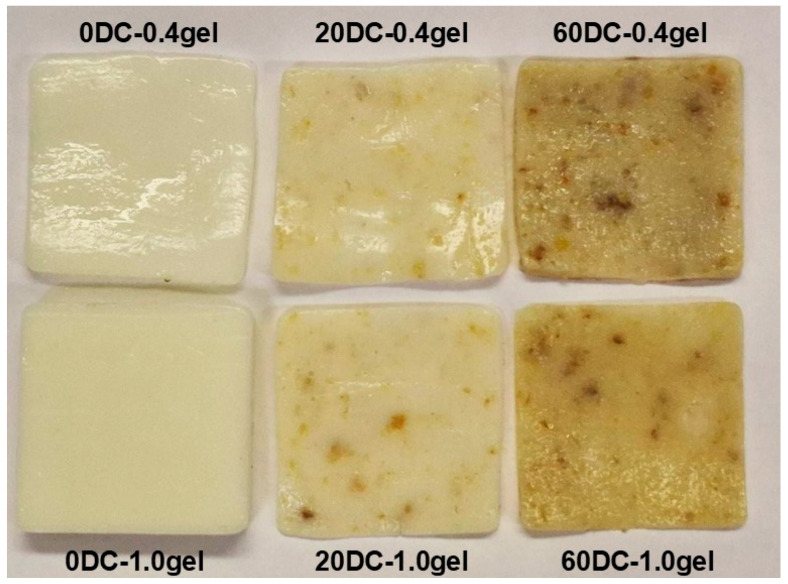
Digital images of κ-carrageenan–konjac gum–milk hydrogels without cells (0DC-0.4gel, 0DC-1.0gel) and encapsulated with 20% (20DC-0.4gel, 20DC-1.0gel) and 60% (60DC-0.4gel, 60DC-1.0gel) carrot callus cells.

Digital images of κ-carrageenan–konjac gum–milk mixed cell-free and carrot callus cell-encapsulated hydrogels are presented in Figure 1. Overall, the distribution of cells and cell conglomerates was uniform throughout all hydrogels. In the 0.4 and 1.0% hydrogels with 20% cells (20DC-0.4gel and 20DC-1.0gel), the number of cells and cell conglomerates was lower than in the hydrogels with 60% cells (60DC-0.4gel and 60DC-1.0gel).

**Figure 2:**
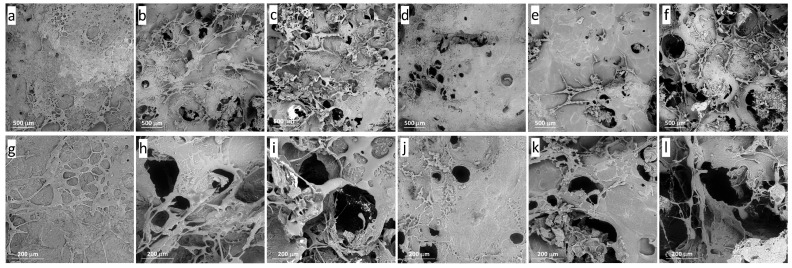
Scanning electron micrographs of κ-carrageenan–konjac gum–milk mixed hydrogels encapsulated with carrot callus cells (DC): cell-free 0DC-0.4gel (**a**,**g**), 20DC-0.4gel (**b**,**h**), 60DC-0.4gel (**c**,**i**), cell-free 0DC-1.0gel (**d**,**j**), 20DC-1.0gel (**e**,**k**), 60DC-1.0gel (**f**,**l**); magnification 63×, scale bar 500 μm (**a**–**f**); magnification 237×, scale bar 200 μm (**g**–**l**).

Scanning electron microphotographs of cross-sections of κ-carrageenan–konjac gum–milk mixed hydrogels encapsulated with carrot callus cells are presented in Figure 2. The cell-free hydrogel (0DC-0.4gel) had an uneven surface with a small number of tiny pores (Figure 2a,g). Increasing the concentration of cells in the hydrogels led to the formation of a more porous microstructure (Figure 2b,c,h,i). The porosity of cell-free (0DC-0.4gel), with 20% cells (20DC-0.4gel), and with 60% cells (60DC-0.4gel) hydrogels was 9.54 ± 2.06, 28.93 ± 3.03, and 40.65 ± 3.12%, respectively (Table 1). A similar trend was shown for 1.0% hydrogels (Figure 2d–f,j–l). The porosity of the 0DC-1.0gel, 20DC-1.0gel, and 60DC-1.0gel hydrogels was 12.03 ± 1.47, 19.78 ± 1.26, and 29.27 ± 2.10%, respectively (Table 1). Overall, 0.4% cell-encapsulated hydrogels were more porous compared to 1.0% cell-encapsulated hydrogels. The formation of a more porous microstructure of the κ-carrageenan–konjac gum–milk hydrogels with cells was likely due to the formation of ice crystals inside the cells during freezing, which led to the formation of pores after the removal of water from the cells during freeze-drying. These data are consistent with our previously obtained data showing that the addition of *Lemna minor* callus cells to pectin and κ-carrageenan hydrogels led to an increase in porosity [13].

The moisture content of the cell-free hydrogels was 83–85% (Table 1). The moisture content of the hydrogels increased with the addition of cells, which was likely related to the presence of cell sap. With an increase in cell concentration to 60% in 0.4 and 1.0% hydrogels, the moisture content increased from 83.81 to 89.91% and from 84.40 to 89.28%, respectively. A similar trend of increased moisture content was observed for 0.5 and 0.7% hydrogels with cells (Table 1).

### 2.2. FTIR Spectroscopy of Hydrogels

**Figure 3:**
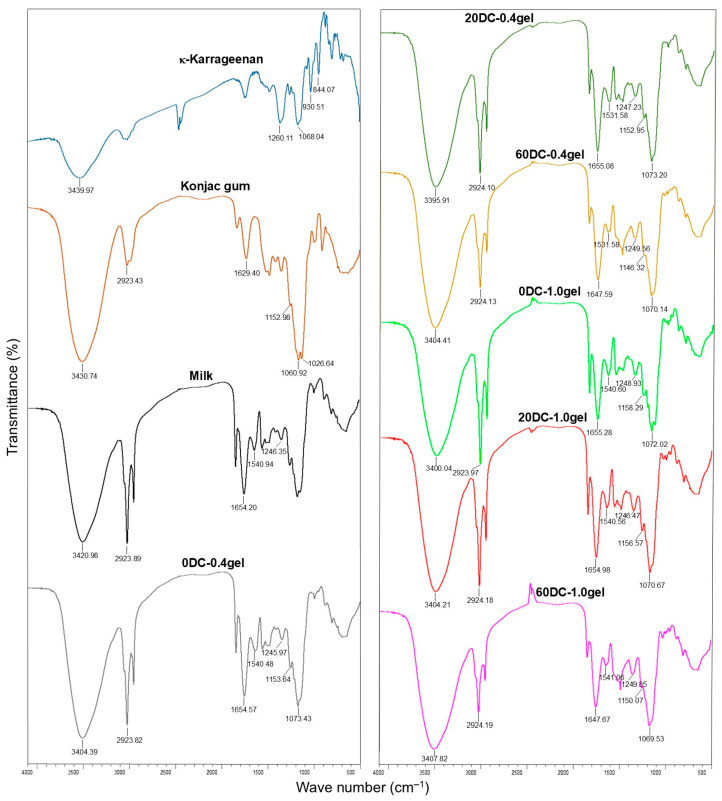
The FTIR spectra of κ-carrageenan, konjac gum, milk, cell-free κ-carrageenan–konjac gum–milk mixed hydrogels (0-DC-0.4gel and 0-DC-1.0gel), and mixed hydrogels encapsulated with carrot callus cells (20-DC-0.4gel, 60-DC-0.4gel, 20-DC-1.0gel, and 60-DC-1.0gel).

The FTIR spectra of κ-carrageenan, konjac gum, milk, cell-free κ-carrageenan–konjac gum–milk hydrogels (0-DC-0.4gel and 0-DC-1.0gel), and hydrogels encapsulated with carrot callus cells (20-DC-0.4gel, 60-DC-0.4gel, 20-DC-1.0gel, and 60-DC-1.0gel) are presented in Figure 3. In the FTIR spectrum of κ-carrageenan, absorption peaks were found at 3439.97 cm^−1^ (–OH stretching vibration), 1260.11 cm^−1^ (ester sulfate groups), 930.51 cm^−1^ (3,6-anhydro-D-galactose), and 844.07 cm^−1^ (D-galactose-4-sulfate) [22,32] (Figure 3). In the FTIR spectrum of konjac gum, peaks were observed at 3430.74 and 2923.43 cm^−1^, corresponding to the –OH and –CH stretching vibrations, respectively [32,33,34] (Figure 3). The peaks at 1629.40 and 1152.98–1026.64 cm^−1^ were assigned to the O–C–O and C–O–C stretching vibrations, respectively [33,34].

In the milk’s FTIR spectrum, absorption bands corresponding to casein were observed. The peaks at 3420.98 and 2923.89 cm^−1^ corresponded to amide A (O–H and N–H stretching) and amide B (=C–H and –NH_3_^+^ stretching), respectively [22,35] (Figure 3). The spectrum had absorption bands at 1654.20 cm^−1^ (amide I, C=O and C–N stretching), 1540.94 cm^−1^ (amide II, C–N and N–H deformation), and 1246.35 cm^−1^ (amide III, C–N stretching and N–H deformation) [27,35].

In the spectra of all mixed hydrogels based on κ-carrageenan, konjac gum, and milk, the wavenumber of the O–H stretching vibrations shifted toward lower wavenumbers, specifically from 3439.97 cm^−1^ for κ-carrageenan, 3430.74 cm^−1^ for konjac glucomannan, and 3420.98 cm^−1^ for milk to 3407.82–3395.91 cm^−1^ for the mixed hydrogels. This indicates an interaction between κ-carrageenan and konjac glucomannan molecules, caused by the formation of hydrogen bonds between the –OSO_3_^–^ groups of κ-carrageenan and the –OH groups of konjac [22,32,36]. Additionally, this indicates an increase in protein-polysaccharide interactions within the composite system and the formation of strong hydrogen bonds [22,27]. Thus, in addition to polysaccharide-polysaccharide interactions, protein-polysaccharide interactions through hydrogen bonds have been demonstrated. In the spectra of all mixed hydrogels, the absorption bands of the ester sulfate groups shifted toward lower wavelengths (1245.97–1249.85 cm^−1^) compared to κ-carrageenan (1260.11 cm^−1^), indicating an interaction between the sulfate groups of carrageenan and the amide groups of milk casein [22].

The spectra of hydrogels with different gel concentrations (0.4 and 1.0%), as well as cells (20 and 60%), were similar, indicating that changing the gel and cell concentrations did not lead to changes or the formation of functional groups. A similar trend was previously shown when the concentration of konjac glucomannan was changed in a whey protein/konjac glucomannan gel [27].

### 2.3. Mechanical Properties of κ-Carrageenan–Konjac Gum–Milk Hydrogels

The mechanical properties of κ-carrageenan–konjac gum–milk/callus hydrogels were investigated by double compression with a texture analyzer. The test simulates the biting action of the mouth, allowing for an understanding of the samples’ behavior during chewing. The following mechanical properties of the hydrogels were investigated: hardness, cohesiveness, gumminess, and springiness.

The hardness of the gel is defined as the peak force that occurs during the first compression cycle and corresponds to the force when compressing a food sample between the molars during the first bite [37,38,39,40]. Cohesiveness characterizes the strength of the internal bonds that support the body of the hydrogel sample [37,39,40], gumminess is the product of hardness and cohesiveness [40], and springiness characterizes the material’s ability to stretch and return to its original height [39].

**Figure 4:**
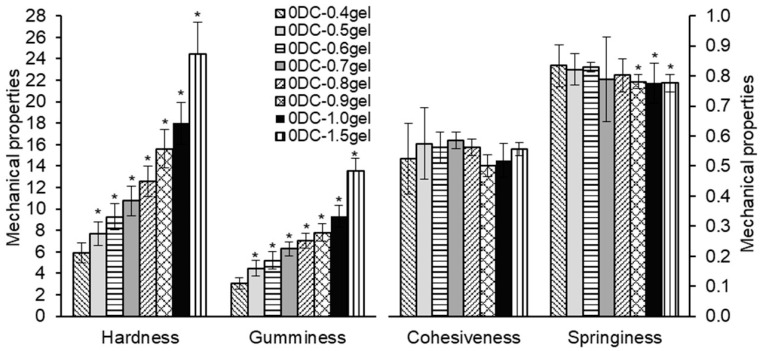
The effect of hydrogel concentration on the mechanical properties of cell-free hydrogels based on κ-carrageenan and konjac gum in a 7:3 ratio and milk. Hardness is expressed in Newtons (N), while gumminess, cohesiveness, and springiness are dimensionless. The data are presented as mean ± SD, *n* = 30–55. * *p* < 0.05 vs. 0DC-0.4gel.

The influence of gel concentration (0.4–1.5%) on the mechanical properties of cell-free hydrogels was investigated. The hardness and gumminess of cell-free hydrogels increased from 5.9 to 24.4 N and from 3.1 to 13.5, respectively, proportional to the increase in gel concentration from 0.4 to 1.5% (Figure 4). A positive correlation was established between hardness, gumminess, and the concentration of hydrogels (r = 0.955 and 0.966, respectively, *p* < 0.001) (Table 2). The effect of hydrogel concentration on gumminess corresponded to that on hardness.

It was previously also shown that the hardness of milk pudding based on κ-carrageenan–konjac mixed gel increased with the increase in gel concentration from 0.1 to 0.3% [23]. Gelation of κ-carrageenan involves a coil-to-helix transition and aggregation of helices through hydrogen bonds, forming a three-dimensional gel structure [41,42,43,44]. Calcium ions present in milk (1.2 mg/mL) interact with the SO_3_^-^ groups of κ-carrageenan, resulting in the cross-linking of helical chains. This leads to the strengthening of the gel network structure. With an increase in κ-carrageenan concentration, the number of helical chains increases, and they are positioned closer to each other, enhancing the cross-linking between the chains [23]. Moreover, the molecules of konjac glucomannan and κ-carrageenan, which are in an ordered helical form, associate through hydrogen bonds [32,44]. The formation of intermolecular hydrogen bonds between κ-carrageenan and konjac glucomannan was confirmed by FTIR spectroscopy (Figure 3). As a result, a denser gel network was formed, and the hardness values of the hydrogel increased.

In the carrageenan–konjac gum system, the network structure was formed by these polysaccharides, and then casein filled this structure. Protein–polysaccharide interactions involve electrostatic forces, hydrogen bonds, and hydrophobic interactions [45]. Among these interactions, electrostatic interactions are the main driving forces [45]. As shown by the FTIR spectroscopy method, the negatively charged sulfate groups of κ-carrageenan interacted electrostatically with the positively charged amino groups of casein, which also contributed to the formation of a denser network structure [22,23] (Figure 3). Therefore, the energy required to break down the hydrogel to a swallowable state increased. Additionally, FTIR spectroscopy has revealed protein–polysaccharide interactions through hydrogen bonds. Previously, hydrogen bonds were detected in casein/κ-carrageenan [22] and whey protein/konjac glucomannan systems [27]. Additionally, as previously demonstrated [27,46], the presence of konjac glucomannan could lead to the restructuring of protein secondary structure, promote the exposure of hydrophobic regions [46], and enhance hydrophobic interactions between proteins and polysaccharides, resulting in an increased degree of intermolecular cross-linking. Thus, the addition of milk to the κ-carrageenan-konjac gum system resulted in a mixed gel characterized by good mechanical properties, which are necessary for further research into the textural and sensory properties of the gels.

A negative correlation was found between springiness and hydrogel concentration (r = −0.306, *p* < 0.001) (Table 2). The decrease in springiness was expressed in the reduced ability of the hydrogel to stretch and return to its original height. According to previously obtained data [23,47], this was likely associated with an increase in κ-carrageenan concentration, leading to greater fragility of the hydrogel. The cohesiveness of hydrogels did not change with the increase in hydrogel concentration (r = −0.012).

**Figure 5:**
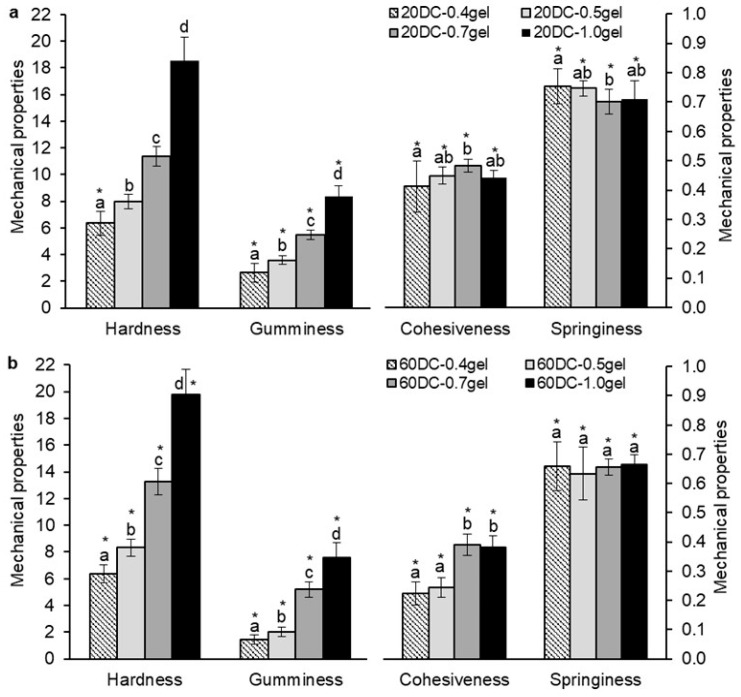
The effect of hydrogel concentration on the mechanical properties of κ-carrageenan–konjac gum–milk hydrogels encapsulated with 20% (**a**) and 60% (**b**) carrot callus cells. Hardness is expressed in Newtons (N), while gumminess, cohesiveness, and springiness are dimensionless. The data are presented as the mean ± S.D., *n* = 30–55. Different lowercase letters (a, b, c, and d) indicate significant differences (*p* < 0.05) between the means for different hydrogel concentrations, * *p* < 0.05 vs. corresponding control (cell-free hydrogels) in Figure 4.

The influence of gel concentration (0.4–1.0%) on the mechanical properties of hydrogels encapsulated with carrot callus cells (20 and 60%) was investigated (Figure 5). The hardness and gumminess of the hydrogels with 20% cells increased proportionally with the increase in gel concentration from 0.4 to 1.0% (Figure 5a). A positive correlation was found between hardness, gumminess, and the concentration of hydrogels (r = 0.975 and 0.967, respectively, *p* < 0.001) (Table 2). Springiness and cohesiveness of hydrogels decreased (r = −0.301, *p* < 0.01) and remained unchanged (r = 0.180, *p* < 0.05), respectively, with increasing hydrogel concentration (Figure 5a).

Hardness (r = 0.977, *p* < 0.001), gumminess (r = 0.955, *p* < 0.001), and cohesiveness (r = 0.792, *p* < 0.001) of hydrogels with high cell content (60%) increased proportionally with the increase in gel concentration from 0.4 to 1.0%, while the springiness of the hydrogels did not change (r = 0.106) (Figure 5b; Table 2). Thus, with increasing gel concentration, the mechanical properties of both cell-free and cell-encapsulated hydrogels were enhanced.

The addition of 20 and 60% carrot callus cells to hydrogels with concentrations of 0.4, 0.5, 0.7, and 1.0% caused a decrease in the gumminess, cohesiveness, and springiness of the hydrogels compared to cell-free hydrogels (Figure 5). The hardness values did not significantly change with the addition of 20% cells compared to the control (Figure 5a). At the same time, the inclusion of a high concentration of cells (60%) in 0.4–1.0% hydrogels led to an increase in hardness by 7.0–23.5% compared to cell-free hydrogels, which was likely due to the contribution of the callus cell hardness [13] (Figure 5b). The decrease in cohesiveness of the hydrogels upon addition of cells was probably due to a decrease in the strength of internal bonds. Plant cells may have interfered with the convergence of carrageenan and/or konjac chains. In addition, the increase in the porosity of the hydrogels upon the addition of cells could also contribute to a decrease in the cohesiveness of the hydrogels. Since gumminess is the product of hardness and cohesiveness [40], its values also tend to decrease with the addition of cells to hydrogels. As the cell concentration increased from 0 to 20% in the 0.4% hydrogels, an increase in hardness by 6.6% was observed (r = 0.180, *p* < 0.05), while further increasing the cell concentration to 60% did not result in an increase in hardness (Table 2 and Table 3).

At the same time, in 1.0% hydrogels, an increase in hardness by 10.0% occurred only at a high cell concentration (60%) (r = 0.393, *p* < 0.001) (Table 2 and Table 3). This was due to the fact that in the weaker 0.4% hydrogels (with a hardness of 5.94 ± 0.93 N), an increase in hardness was observed due to the contribution of callus cell hardness. The stronger 1.0% hydrogels initially had a high hardness (18.00 ± 1.97 N), and adding cells at a lower concentration (20%) did not significantly affect their hardness. However, adding a higher concentration of cells (60%) to the 1.0% hydrogel resulted in a more significant increase in hardness. This was because the hardness of a larger number of cells contributed to the overall hardness of the hydrogel. Gumminess, cohesiveness, and springiness decreased with the increase in cell concentration in the 0.4 and 1.0 hydrogels (Table 3). A negative correlation was found between gumminess (r = −0.782, −0.579, *p* < 0.001), cohesiveness (r = −0.813, −0.765, *p* < 0.05), and springiness (r = −0.702, −0.600, *p* < 0.001) of 0.4 and 1.0% hydrogels, respectively, and the cell concentration in them (Table 2). The increase in cell concentration in the hydrogels likely led to a more significant decrease in the strength of the internal bonds and an increase in the porosity of the hydrogels, which resulted in a reduction in their cohesiveness and gumminess. Our results are consistent with previously obtained data for κ-carrageenan hydrogels enriched with LA14 lupine callus tissue [9]. It was shown that the hardness of such gels increased, while their cohesiveness and springiness decreased with an increasing content of callus cells in them [9].

When encapsulating cells in hydrogels, a more significant reduction in mechanical properties such as gumminess, cohesiveness, and springiness was observed for the weaker 0.4% hydrogels compared to the 1.0% hydrogels. Gumminess, cohesiveness, and springiness decreased compared to cell-free hydrogels by 1.2–2.1, 1.3–2.3, and 1.1–1.3 times, respectively, for 0.4% hydrogels, and by 1.2, 1.2–1.4, and 1.1–1.2 times, respectively, for 1.0% hydrogels. This was due to the fact that when the concentration of κ-carrageenan and konjac gum was reduced to 0.4%, a less dense gel network and a more porous microstructure of the hydrogel were formed.

The stability of the mechanical properties of the hydrogels was investigated after the hydrogels were stored at 4 °C for 5 days. It was shown that the hardness of the cell-free hydrogel 0DC-0.4gel did not change after 5 days, while the hardness of the cell-containing hydrogels 20DC-0.4gel and 60DC-0.4gel increased by 23.4% and 36.8%, respectively. The cohesiveness of 0DC-0.4gel, 20DC-0.4gel, and 60DC-0.4gel hydrogels increased by 32.7%, 31.7%, and 72.7%, respectively, after 5 days. The gumminess of 0DC-0.4gel, 20DC-0.4gel, and 60DC-0.4gel hydrogels increased by 12.5%, 39.4%, and 133.3%, respectively, after 5 days. The springiness of the 0DC-0.4gel and 20DC-0.4gel hydrogels did not change, while that of the 60DC-0.4gel increased by 6.1%. A more significant increase in hardness, cohesiveness, gumminess, and springiness after 5 days was observed in hydrogels with a high cell content (60DC-0.4gel). This is likely due to the fact that the initial high-cell-concentration gels (60%) contained more moisture compared to the cell-free gels and the gels with 20% cells (Table 1), and these gels underwent greater dehydration due to the evaporation of more moisture. A similar trend in the change of mechanical characteristics was observed for both 1.0% hydrogels with and without cells.

### 2.4. Rheological Properties of Hydrogels

**Figure 6:**
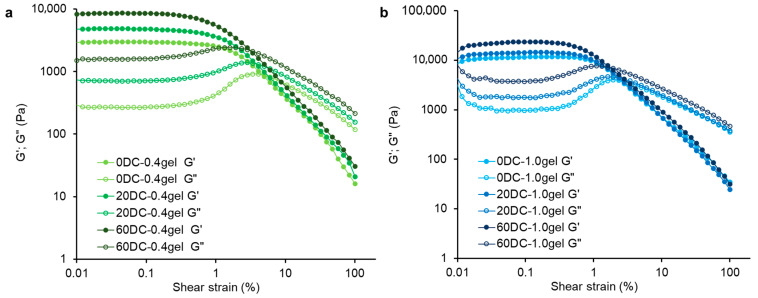
Rheological properties of 0.4% (**a**) and 1.0% (**b**) κ-carrageenan–konjac gum–milk hydrogels. Storage modulus (G′, filled symbols) and loss modulus (G”, empty symbols) are represented as a function of shear strain at fixed frequency of 1 Hz and 20 °C.

For the study of the rheological characteristics of hydrogels, the softest (0.4%) and harder (1.0%) gels were selected, for both those encapsulated with cells (20DC-0.4gel, 60DC-0.4gel, 20DC-1.0gel, and 60DC-1.0gel) and cell-free hydrogels (0DC-0.4gel, 0DC-1.0gel). The storage modulus G’ was significantly higher than the loss modulus G” in the LVE region for all hydrogel samples, indicating ideal elastic gel-like characteristics of the samples (Figure 6, Table 4). The G’ and G” values in the LVE region were higher for 1.0% hydrogels than for 0.4% hydrogels, indicating a higher gel network strength of 1.0% hydrogels. Previously, it was shown that in casein/κ-carrageenan gels, G’ and G’’ increased with increasing κ-carrageenan concentration [22]. Adding cells to the hydrogels led to an increase in G’ and G” in the LVE region, indicating an increase in the strength of the gel network of the cell-encapsulated gels. As the cell concentration increased from 20 to 60%, the values of G’ and G” increased by 1.8–2.3 and 1.6–2.1 times for the 0.4 and 1.0% hydrogels, respectively (Table 4). The rheological characteristics are consistent with the mechanical properties, particularly the increase in hydrogel hardness with increasing gel and cell concentration. The obtained data are consistent with previously obtained data, which showed that incorporating carrot callus cells into weak, low-hardness xanthan/konjac gums hydrogels [12] and the inclusion of thawed lupine callus tissue in κ-carrageenan gels [9], as well as increasing the cell concentration in hydrogels, led to an increase in the values of G’ and G”.

The loss tangent Tan [δ]_LVE_ for all hydrogels was 0.10–0.19, indicating solid-like behavior of the samples (Table 4). The slope of the loss tangent in the nonlinear region Tan [δ]_AF_ was similar for all hydrogels, indicating similar hydrogel spreadability (Table 4). The fracture stress (τFr) indicates the transition point of the hydrogel from an elastic to a viscous state. The τFr values for the 1.0% gels were higher than for the 0.4% gels, indicating greater resistance to shear stress. The fracture strain (γFr) values for all 0.4% gels were higher than for the 1.0% gels, indicating greater fragility of the 1.0% gels. The increase in hydrogel fragility was likely related to the increase in the concentration of κ-carrageenan [23,47].

### 2.5. Sensory Properties and Chewing of Hydrogels

Innovative food gel products that incorporate plant tissue cultures require an acceptability assessment, which is commonly evaluated using liking metrics [19,48]. Therefore, first, overall and consistency liking were rated by 31 nontrained volunteers using a nine-point hedonic scale to exclude the negative impact of callus cells on the consumer’s acceptability of the gel.

The addition of 60% carrot callus cells in 0.4% hydrogel decreased overall and consistency liking by 25% and 24%, respectively (4.0 ± 1.6 vs. 5.3 ± 1.5 and 4.1 ± 1.3 vs. 5.4 ± 1.5, both *p* < 0.001). Liking scores of 1.0% hydrogels were independent of the cell content. The Pearson correlation coefficient (r) for the dependence of the overall liking ratings on the cell concentration was −0.34 (*p* < 0.001) and −0.19 (n.s.) for 0.4 and 1.0% hydrogels, respectively. A high correlation coefficient (r = 0.74, *p* < 0.001) was found between overall liking and consistency liking, confirming that texture is important for hedonic evaluation. Overall liking positively correlated with instrumental cohesiveness and springiness (r = 0.27 and 0.25, respectively; both *p* < 0.001), and it did not correlate with instrumental hardness. The data differ from studies that showed that increasing the hardness of model food gels or gel-based food increased [16,49] or decreased [50,51] overall acceptability and liking ratings. On the other hand, several studies reported that modification of product hardness with hydrocolloids did not affect hedonic ratings [52,53,54,55]. The lack of an increase in overall liking appears to be a useful feature when developing functional foods for overweight individuals. An increase in the hedonic effect could lead to increased consumption of the gel product, which would be counterproductive to the goal of reducing food intake.

Then, volunteers used a 100 mm visual analog scale to rate the perceived intensity of textural qualities of the gels. Textural qualities are usually grouped into three main classes: mechanical, geometric, and other attributes, such as moisture [56]. Perceived ‘hardness,’ ‘springiness,’ and ‘adhesiveness’ were examined as mechanical parameters of the initial and mastication stages of oral processing, respectively. The subjective ratings of “swallowability” and “juiciness” were selected to describe the bolus’s preparedness for swallowing as well as the moisture release during mastication [57]. Graininess, as a geometric attribute exhibited during mastication, is associated with the size, shape, and number of particles embedded in semi-solid and liquid foods. It is usually induced by the contact between tongue and palate when they rub against food particles [58] and was assumed to change when perceiving a gel containing callus cells.

The perceived hardness of 1.0% hydrogels was higher than that of 0.4% hydrogels (Table 5). Food’s hardness is one of its most noticeable sensory characteristics. Perception of hardness occurs at the initial phase of mastication, reflecting the force needed to cause a food deformation upon the first chew [38]. As expected, perceived hardness was found to be positively correlated with instrumental hardness and gumminess (Table 5). Furthermore, volunteers perceived the springiness of the 1.0% hydrogel as higher than that of the 0.4% hydrogel. ‘Springiness’ refers to the sensations that occur while compressing a sample between the tongue and hard palate during the initial phase of oral processing, and it is defined as the time necessary for a food to recover from deformation after unloading [57]. Perceived and instrumental springiness were not correlated, while perceived springiness was correlated with instrumental hardness and gumminess (Table 5). Swallowability and juiciness decreased, while adhesiveness remained unchanged with increasing gel concentration (Table 5). Adhesiveness, swallowability, and juiciness scores had no or weak interrelationships with the mechanical properties of hydrogels.

Using Pearson’s coefficient (r), perceived hardness was found to be correlated with instrumental hardness and gumminess (Table 6). Perceived and instrumental springiness were not correlated, while perceived springiness was correlated with instrumental hardness and gumminess. Adhesiveness, swallowability, and juiciness scores had no or weak interrelationships with the mechanical properties of hydrogels.

The addition of 60% carrot callus cells to 0.4% hydrogel increased perceived hardness and adhesiveness by 2.8-fold and 1.8-fold, respectively (Table 6). The cells did not affect subjective ratings of hardness for the 1.0% hydrogel and increased the perceived adhesiveness by 1.4-fold. ‘Adhesiveness’ relates to primary mechanical parameters and is defined as the amount of labor necessary to overcome the adhesion force formed between the food bolus and the surfaces in the oral cavity with which it comes into contact [38]. Adhesive foods have slower eating rates because they exhibit elastic behavior while attaching to oral surfaces, making it more difficult to agglomerate bolus particles into a bolus [59]. Therefore, obese people may be a potential group of consumers of callus cell gels, as they need to reduce their eating rate. In addition, the gels we obtained can be useful for people with a dry mouth diagnosis. Xerostomia, or “dry mouth,” affects around 20% of the general population, and adhesive agents are widely used for symptomatic treatments of dry mouth [60]. The adhesiveness of the studied gels may be partially due to the mucoadhesive properties of κ-carrageenan, which is a polyanion. However, the addition of callus cells further increased the adhesiveness. The perception of adhesiveness is determined by a combination of both the surface and rheological properties of the food bolus and the lubricating ability of saliva. Why the addition of plant cells increased the feeling of adhesiveness remains unclear. The addition of carrot callus cells did not affect the springiness, swallowability, or juiciness ratings of the hydrogels.

**Figure 7:**
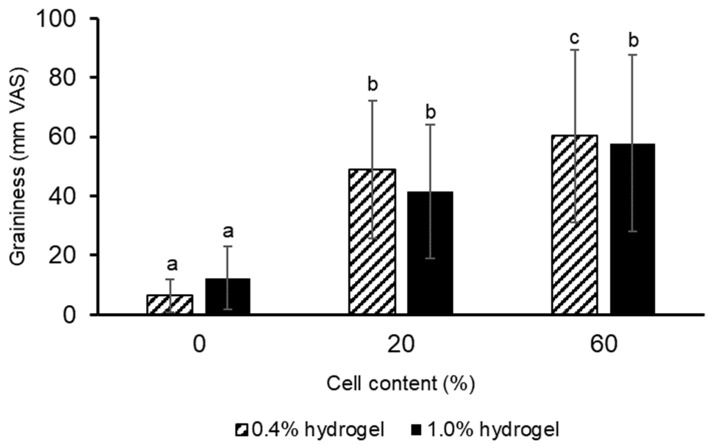
The graininess perception by volunteers of 0.4% and 1.0% κ-carrageenan–konjac gum–milk hydrogels enriched with 0, 20, and 60% of carrot callus cells. The data are presented as the mean ± S.D. ^a, b, c^—*p* < 0.05 between different letters (*n* = 31).

Volunteers assessed the graininess of hydrogels without carrot callus cells as minor, measuring 6.4 ± 5.6 and 12.3 ± 10.6 mm on a 100 mm visual scale for 0.4 and 1.0% hydrogels, respectively (Figure 7). The addition of 20% plant cells in the hydrogel increased the graininess scores of the 0.4% and 1.0% hydrogels by 7.7 and 3.5 times, respectively. An increase in cell content to 60% enhanced the graininess scores of the 0.4% hydrogel by 22%. However, the graininess assessments of 1.0% hydrogels containing 20 and 60% plant cells did not differ. The differences in graininess ratings for 0.4 and 1.0% gels can be explained by the fact that gels with low hardness are broken down into many small particles and are perceived as grainy towards the end of oral processing. At the end of the sensory trajectory, it was previously demonstrated that gels are divided into two groups: one set of high fracture strain gels that are regarded as creamy and another group of low fracture strain gels that are perceived as grainy [61].

The Pearson correlation coefficient for the dependence of the graininess ratings on the cell concentration was 0.63 and 0.61 (both *p* < 0.001) for 0.4 and 1.0% hydrogels, respectively. The graininess ratings were negatively correlated with elasticity (r = −0.64, *p* < 0.001) and cohesiveness (r = −0.60, *p* < 0.001) but were not associated with the instrumental hardness and gumminess.

It is well acknowledged that determining the graininess of food is a very complicated process that can hardly be appreciated using an instrumental approach. The size, shape, and quantity of particles embedded in semi-solid and liquid foods are specifically linked to the texture trait known as graininess [58]. Previously [62] showed that the agar and gelatine emulsion-filled gels with a high concentration of gelling agent resulted in an increased graininess. Shewan, Stokes, and Smyth [63], using samples containing spherical microgels, found that increasing particle modulus would cause an increase in graininess perception. It is well recognized that the human mouth typically uses tribological motions to manipulate food with the tongue and palate, resulting in an overall graininess [64]. The oral tactile system serves as the primary sensory source for the perception of food graininess, with a detection threshold for graininess in humans likely around 30 μm for chocolates [65]. The average diameter of carrot callus cells was 88.0 ± 16.4 μm; therefore, a grainy sensation was expected when adding carrot callus cells to the hydrogel.

The interrelationships between the textural qualities are presented in Table 7. Ratings of hardness and springiness were positively correlated, while swallowability was inversely associated with hardness, springiness, and adhesiveness. Among sensory attributes, the graininess scores were only positively correlated with the juiciness of the hydrogels.

The mastication of κ-carrageenan–konjac gum–milk hydrogels was influenced by hydrogel hardness and carrot callus cell content, according to electromyography (EMG) experiments. Chewing 0.4% hydrogel to a ready-to-swallow state required less time, fewer chews, and less masticatory muscle activity than chewing 1.0% hydrogel (Table 8). Adding 20% cells to 0.4% hydrogel increased the activity of the masseter and temporalis muscles by 33% and had no effect on the activity of the suprahyoid muscles. Increasing the cell concentration to 60% increased the activity of the temporalis muscle during gel chewing, while the activity of the masseter and suprahyoid muscles was unchanged. Chewing the hydrogel containing 60% cells increased the masseter, temporalis, and suprahyoid muscles’ activity by 29, 42, and 15%, respectively, compared to chewing the cell-free hydrogel.

It is known that masticatory behavior is determined by individual characteristics, including differences in overweight and obese adults compared to normal-weight adults [66]. Therefore, we next analyzed sensory perception and chewing in individuals with normal (22.3 ± 1.4 kg/m^2^, *n* = 17) and elevated (28.3 ± 2.0 kg/m^2^, *n* = 12) body mass index (BMI), identified among our volunteers.

**Figure 8:**
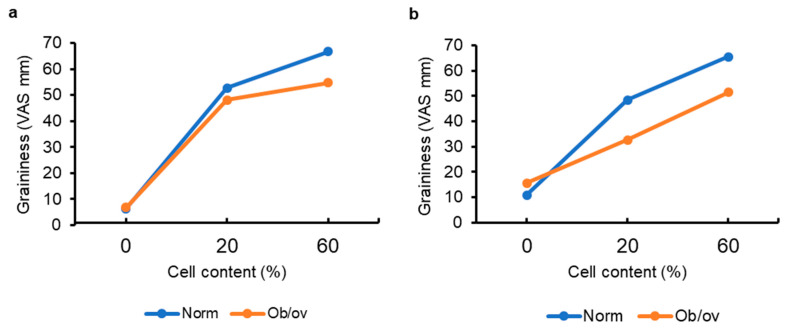
The graininess perception of 0.4% (**a**) and 1.0% (**b**) κ-carrageenan–konjac gum–milk hydrogels enriched with 0, 20, and 60% of carrot callus cells. Norm and Ob/ov represent individuals with normal (22.3 ± 1.4 kg/m^2^, *n* = 17) and elevated (28.3 ± 2.0 kg/m^2^, *n* = 12) body mass index, respectively. The data are presented as the mean ± S.D.

The graininess score of 0.4% hydrogel increased with increasing cell content to the same extent, regardless of BMI. Namely, with an increase in cell content from 0 to 60%, the graininess score increased by 60.3 ± 24.5 and 47.8 ± 36.4 mm VAS (*p* = 0.364) in the subgroups with normal and elevated BMI, respectively (Figure 8). The graininess score of 1.0% hydrogel increased with increasing cell content from 0 to 60% by 54.6 ± 26 and 35.9 ± 36.2 mm VAS (*p* = 0.048) in the subgroups with normal and elevated BMI, respectively.

Food texture has been demonstrated to have a substantial influence on eating habits and body weight [67]. Even little variations in graininess affect eating rate. For example, reducing the particle size of granola added to yogurt from 12 mm to 6 mm at a constant weight concentration during ad libitum consumption boosted eating rate and intake by 5% and 7%, respectively, without influencing liking [68]. The data obtained suggest that people with elevated BMI have lower sensitivity to graininess than people with normal BMI. It is known that human oral tactile functions are mediated by mechanoreceptors in the oral mucosa; hence, it is reasonable to anticipate that the functionality of oral mechanoreceptors is altered in people with high BMI. Previously, the altered taste sensitivity was reported in individuals with a higher BMI [69]. In a study [70], sensory properties and acceptability of a model custard dessert of different textures were investigated in normal-weight and obese women. Only obese women were found to perceive the increase in creaminess of desserts and to give a higher creaminess score than normal-weight subjects. Obese women liked the samples with the addition of xanthan gum significantly more than normal-weight women, demonstrating that BMI may influence the perception of textural qualities. In a subsequent study, the same group of authors found no dependence on the creaminess rating of pudding samples, differing in the concentration of the thickening agent [71]. The study in [72] also did not reveal a relationship between texture sensitivity and BMI, but this conclusion was based on the results of a self-reported questionnaire.

When tasting 60DC-1.0 hydrogel, volunteers with elevated BMI rated the swallowability 43% higher than subjects with normal body weight (54 ± 27 and 77 ± 29 mm VAS, *p* = 0.02). As is generally accepted, increasing the rate of eating decreases satiation, increases caloric intake, and is therefore a strong risk factor for obesity [67]. It could be assumed that the perception of the gel sample as easier to swallow could contribute to its faster consumption. The perception of other hydrogel samples and other textural qualities did not differ between subjects with different BMIs.

Contrary to our above assumption, the duration and frequency of hydrogel chewing were independent of BMI. However, EMG signal amplitude and activity of suprahyoid muscles were 29–35% lower in volunteers with elevated BMIs than in those with normal BMIs (Table 9). These EMG data were consistent with the sensory assessment data. It is logical that the easier-to-swallow gel stimulated less activity in the suprahyoid muscles. Masseter and temporalis muscle activities during hydrogel chewing were independent of BMI. The obtained data are consistent with studies that have identified an association between chewing efficiency, chewing parameters, masticatory muscle activity, and BMI [73,74]. However, it should be noted that attenuation of EMG signals may be due to the fact that anhydrous adipose tissue lying underneath the skin layer is a poorer conductor than muscle. It has previously been shown that obese individuals tend to have much lower amplitudes than thin individuals [75]. The amount of subcutaneous fat is typically thought to be strongly related to total body fat; however, we did not quantify the amount of subcutaneous fat on the volunteers’ faces, which is a study limitation.

Another drawback of the study was the lack of comparison with a conventional gel system during sensory and physiological (EMG) assessment of the prepared gels. The results obtained have limited practical value, as they do not demonstrate the benefits or drawbacks of κ-carrageenan–konjac gum–milk hydrogels loaded with callus cells.

## 3. Conclusions

An encapsulation of 20 and 60% carrot callus cells in 0.4 and 1.0% κ-carrageenan–konjac gum–milk mixed hydrogels increased their mechanical hardness by 7–10%. The springiness, cohesiveness, and gumminess of the hydrogels decreased with an increase in the plant cell concentration from 20 to 60%. Adding cells to the hydrogels led to an increase in the storage modulus G’ and the loss modulus G”, indicating an increase in the gel network strength. With an increase in cell concentration, the G’ and G” values increased.

The overall liking of the hydrogels did not change with the addition of cells, except for the liking scores of the 0.4% hydrogel containing 60% carrot callus cells, which decreased. The addition of 60% carrot callus cells to 0.4% hydrogel increased perceived hardness and adhesiveness by 2.8-fold and 1.8-fold, respectively. The cells did not affect subjective ratings of hardness for the 1.0% hydrogel and increased the perceived adhesiveness by 1.4-fold. The addition of 20% plant cells in the hydrogel increased the graininess scores of the 0.4% and 1.0% hydrogels by 7.7 and 3.5 times, respectively. An increase in cell content to 60% enhanced the graininess scores of the 0.4% hydrogel by 22%. However, the graininess assessments of 1.0% hydrogels containing 20 and 60% plant cells did not differ. The graininess ratings were positively correlated with the cell concentration and negatively correlated with springiness and cohesiveness. The influence of cells on the graininess score was smaller in volunteers with elevated BMI compared to ones with normal weight. However, volunteers with elevated BMI rated the swallowability higher than subjects with normal body weight. EMG experiments revealed that adding 60% cells increased the activity of the masseter and temporalis muscles during gel chewing by 21–29 and 42–53%, respectively. Moreover, suprahyoid muscle activity was lower in volunteers with elevated BMIs than in those with normal BMIs. This suggests individual variability in oral processing and sensory perception, which must be taken into account when determining textural qualities of grainy gels containing plant cells.

Thus, the incorporation of plant cells into gum hydrogel represents a promising approach to creating unique gel textures and developing innovative functional foods. Obese people may be a potential group of consumers of gels with callus cells, since the increased hardness and adhesiveness of the gels may result in a decrease in the rate of eating. Further studies on the effects of callus cell gels on satiety and food intake would be promising in this regard.

## 4. Materials and Methods

### 4.1. Materials

Konjac gum was purchased from Foodchem International Corporation, Shanghai, China. Konjac gum, obtained from the tubers of the Amorphophallus konjac plant, is a glucomannan consisting of a linear chain of 1,4-linked D-mannose and D-glucose residues in a ratio of 1.6:1, with a viscosity of 3700 mPa·s. κ-Carrageenan was purchased from Green Fresh Foodstuff Co., Ltd., Zhangzhou City, Fujian, China. Monosulfated κ-carrageenan, obtained from red seaweed, consisted of an alternating linear chain of α(1→3)-D-galactose-4-sulfate and β(1→4)-3,6-anhydro-D-galactose. The molecular weight of κ-carrageenan was 1670 kDa, and the number of sulfate groups was 11.7%. Kinetin and naphthylacetic acid were obtained from Serva, Heidelberg, Germany. Fresh milk (whole milk) with a fat mass fraction of 3.2% was taken from a supermarket (Syktyvkar Dairy Plant, Syktyvkar, Russia). The protein, fat, and carbohydrate concentrations in 100 g of milk were 3.0, 3.2, and 4.7 g, respectively. The calcium ion concentration in the milk was 1.2 mg/mL. All other chemicals were of analytical grade.

### 4.2. Callus Culture of Daucus Carota

The callus culture of carrots, *Daucus carota* subsp. sativus (Hoffm.) Arcang, obtained in the Institute of Physiology of the Federal Research Center “Komi Science Center of the Urals Branch of the Russian Academy of Sciences”, was maintained at a temperature of 24 °C and a humidity of 60% in a thermostat in the dark with an interval of 28 days. Callus was cultivated on modified Murashige and Skoog’s medium [76] containing kinetin (0.1 mg/L) and naphthylacetic acid (1.0 mg/L). Carrot callus cells obtained from the root vegetable were orange-colored, rounded parenchymal cells with an average diameter of 88.0 ± 16.4 μm. Non-viable carrot callus cells were used in the study. This is because the gel preparation process involved heat treatment at 80 °C, during which plant cells lost their viability. The viability of carrot callus cells after encapsulation in hydrogel was determined using the Evans blue exclusion staining method [4].

### 4.3. Preparation of κ-Carrageenan–Konjac Gum–Milk Hydrogels Encapsulated with Callus Cells

In a preliminary study to prepare κ-carrageenan–konjac gum–milk hydrogels, κ-carrageenan and konjac gum were mixed in ratios of 1:1, 3:7, and 7:3 (*w*/*w*). The mechanical characteristics of the resulting gels were determined, based on which the optimal polysaccharide ratio was selected. The ratio of κ-carrageenan to konjac gum of 7:3 (*w*/*w*) was chosen for preparing the gels because it resulted in gels with the best mechanical characteristics (hardness, gumminess, springiness, and cohesiveness).

κ-Carrageenan and konjac gum were mixed in a ratio of 7:3 (*w*/*w*), and the total concentration of the mixture in milk was 0.4, 0.5, 0.6, 0.7, 0.8, 0.9, 1.0, and 1.5%. The mixture of κ-carrageenan and konjac gum was added to whole milk, then stirred on a magnetic stirrer at 80 °C for 30 min and kept in a water bath at 80 °C for 30 min. The resulting mixture was placed in molds (size 24 × 24 mm and height 9 mm) and kept at 10 °C for 20 h. After that, the hydrogel samples were cut into pieces (size 12 × 12 mm and height 9 mm). The obtained cell-free hydrogels (0DC-0.4gel–0DC-1.5gel) served as controls.

We preliminarily investigated the effect of carrot callus (DC) cell concentration range (10, 20, 40, 60, and 80%) on the mechanical properties of the gels. Adding a higher concentration of cells (80%) made it difficult to obtain a gel, as the cells interfered with the formation of the gel network. At the same time, the very low cell concentration (10%) made it difficult to identify patterns in the effect of cell addition on the mechanical and sensory characteristics of the gels. Cell concentrations of 20% and 60% were chosen for further research because this allowed us to identify the effect of low and high cell concentrations on the mechanical, rheological, and sensory properties of the gels.

To obtain hydrogels encapsulated with carrot callus cells, cells were added to 0.4, 0.5, 0.7, and 1.0% hydrogels at concentrations of 20 and 60% (*w*/*w*). The mixture was stirred on a magnetic stirrer at 80 °C for 30 min and kept in a water bath at 80 °C for 30 min. Then, hydrogel samples were prepared as described above for cell-free samples. The composition of the hydrogels is presented in Table 1. For subsequent study of the sensory properties and chewing of the hydrogels, the softest gels (0.4%) and the harder gels (1.0%) were selected.

### 4.4. Scanning Electron Microscopy

The hydrogels were freeze-dried at the ice condenser temperature of −55 °C and the pressure of 0.021 mbar using a Beta 2–8 LD plus (Martin Christ, Osterode am Harz, Germany). The dried hydrogel samples were coated with gold using a sputtering device (DSCR, Nano-Structured Coatings Company, Tehran, Iran). The microstructure of the hydrogels was determined using a scanning electron microscope (SEM) (Tescan Vega3 SBU, Brno, Czech Republic) at 20 kV. Magnifications of 63× (scale 500 μm) and 237× (scale 200 μm) were used to obtain SEM images.

### 4.5. Porosity Measurement

To determine the porosity of hydrogels, the solvent replacement method was used [77]. Samples of freeze-dried hydrogels were immersed in absolute ethanol overnight, then excess ethanol was removed from the surface, and the samples were weighed on an analytical balance. Porosity was calculated using the formula: Porosity (%) = (M2 − M1)/(ρV) × 100, where M1 and M2 are the masses of the hydrogel before and after ethanol immersion, respectively, ρ is the density of absolute ethanol, and V is the volume of the hydrogel. The tests were performed in 8–12 replicates.

### 4.6. The Moisture Content

To determine the moisture content in the hydrogels, wet samples were weighed on an analytical balance, then freeze-dried, and weighed again. The moisture content in the hydrogels was determined according to the equation: MC (%) = 100 − M2 × 100/M1, where M1 and M2 are the masses of the hydrogel before and after freeze-drying, respectively. The tests were performed in 8–12 replicates.

### 4.7. FTIR Spectroscopy

Infrared Fourier transform spectroscopy (FTIR) of κ-carrageenan, konjac gum, milk, cell-free κ-carrageenan–konjac gum–milk hydrogels, and hydrogels encapsulated with carrot callus cells was performed using a FSM2202 FTIR spectrometer (Infraspec, Saint Petersburg, Russia). Lyophilized samples were pre-ground, mixed with KBr, and pressed. The samples were scanned at a wavelength range of 4000 to 400 cm^−1^ with a resolution of 4 cm^−1^.

### 4.8. Mechanical Properties of Hydrogels

The mechanical properties of κ-carrageenan–konjac gum–milk/callus hydrogels were investigated using a Texture Analyzer (TA-XT Plus, Texture Technologies Corp., Stable Micro Systems, Godalming, UK). A double-compression test was performed on hydrogel samples (9 mm high, 12 mm long, and 12 mm wide). The hydrogel samples were compressed twice using a P/25 cylindrical aluminum probe (25 mm in diameter) at a test speed of 1 mm/s to 100% strain at 23 °C. Thirty to fifty-five replicates were performed for different hydrogel samples. Using Texture Exponent 6.1.4.0 software (Stable Micro Systems, Godalming, UK), the parameters such as hardness, cohesiveness, gumminess, and springiness were investigated.

The stability of the mechanical properties of the hydrogels was investigated after the hydrogels were stored at 4 °C for 5 days. The percentage changes in the mechanical characteristics (hardness, cohesiveness, gumminess, and springiness) compared to the initial hydrogels, taken as 100%, were determined.

### 4.9. Rheological Properties

For strain sweep measurements, a rotational rheometer (Anton Paar, Physica MCR 302, Graz, Austria) was used, which was equipped with parallel geometry plates (25 mm diameter, 1 mm gap). The amplitude sweep measurements of the hydrogels covered the strain range from 0.01 to 100% at a constant frequency of 1 Hz and 20 °C using the controlled shear rate mode. The storage modulus (G’_LVE_), loss modulus (G”_LVE_), loss tangent (Tan [δ]_LVE_), and slope of the loss tangent after the yield point (Tan [δ]_AF_), the fracture stress (τFr), and the fracture strain (γFr) were determined as described in [78].

### 4.10. Sensory Evaluation and EMG Activity

The Bioethics Committee of the Institute of Physiology of the Federal Research Centre “Komi Science Center of the Urals Branch of the Russian Academy of Sciences” has accepted the study protocol (approval no. 10/10 March 2022). Thirty-one volunteers signed informed consent and filled out a participant questionnaire that included their gender, age, height, and weight.

Three samples of each hydrogel were prepared the day before, stored overnight at 4 °C, and warmed to room temperature immediately before testing. The participants were asked to score the overall liking and consistency of the first hydrogel sample from extremely disliked (1 point) to extremely liked (9 points). Then, participants rated the taste, odor, hardness, springiness, adhesiveness, swallowability, and juiciness of the second sample according to [56] using a 100 mm visual analogue scale. Then, EMG activity from the masseter, temporal muscles, and suprahyoid muscles was determined by surface EMG recording during unilateral chewing of the third hydrogel sample as described earlier [19].

### 4.11. Statistical Analysis

To determine the differences in the mechanical characteristics of the hydrogels, one-way ANOVA including Tukey’s HSD test was used. Statistical differences were considered significant at *p* < 0.05. The obtained data are presented as the mean ± standard deviation (S.D.). Using Microsoft Excel 2019, correlation coefficients were determined, and their significance was assessed to identify the relationship between various parameters. Normality of data distribution was determined by the Shapiro–Wilk test. Since the data had a non-normal distribution, the Friedman’s test and the Durbin post hoc test were used to compare the mean values of sensory and EMG data obtained during the chewing of different food gels.

## Figures and Tables

**Table 1:** Characterization of κ-carrageenan–konjac gum–milk hydrogels.

Gel Formulation	Concentration of Gel (%)	Concentration of DC Callus Cells (%)	Porosity (%) (*n* = 8–12)	Moisture Content (%) (*n* = 8–12)
0DC-0.4gel	0.4	0	9.54 ± 2.06	83.81 ± 0.3
0DC-0.5gel	0.5	0	13.93 ± 1.21	84.73 ± 1.1
0DC-0.6gel	0.6	0	13.60 ± 1.49	84.06 ± 0.71
0DC-0.7gel	0.7	0	11.04 ± 1.32	83.20 ± 0.76
0DC-0.8gel	0.8	0	11.64 ± 1.04	84.57 ± 1.25
0DC-0.9gel	0.9	0	11.98 ± 2.07	84.69 ± 0.04
0DC-1.0gel	1.0	0	12.03 ± 1.47	84.40 ± 0.63
0DC-1.5gel	1.5	0	11.90 ± 0.56	83.06 ± 0.64
20DC-0.4gel	0.4	20	28.93 ± 3.03 ^a^	85.40 ± 0.63 ^a^
60DC-0.4gel	0.4	60	40.65 ± 3.12 ^a^	89.91 ± 0.34 ^a^
20DC-0.5gel	0.5	20	28.69 ± 2.80 ^b^	86.10 ± 0.97 ^b^
60DC-0.5gel	0.5	60	33.43 ± 2.18 ^b^	90.23 ± 0.72 ^b^
20DC-0.7gel	0.7	20	26.18 ± 1.39 ^c^	85.72 ± 0.31 ^c^
60DC-0.7gel	0.7	60	28.00 ± 1.11 ^c^	89.45 ± 0.16 ^c^
20DC-1.0gel	1.0	20	19.78 ± 1.26 ^d^	85.97 ± 0.57 ^d^
60DC-1.0gel	1.0	60	29.27 ± 2.10 ^d^	89.28 ± 0.15 ^d^

The data are presented as the mean ± S.D. DC—cells of *Daucus carota* callus culture. a, *p* < 0.05 vs. 0DC-0.4gel; b, *p* < 0.05 vs. 0DC-0.5gel; c, *p* < 0.05 vs. 0DC-0.7gel; d, *p* < 0.05 vs. 0DC-1.0gel.

**Table 2:** Correlation between the mechanical properties of hydrogels, hydrogel concentration, and cell content in the hydrogel.

Mechanical Properties	Concentration of Cell-Free Hydrogel	Concentration of Hydrogel with 20% Cells	Concentration of Hydrogel with 60% Cells	Cell Content in 0.4% Hydrogel	Cell Content in 1.0% Hydrogel
Hardness (N)	0.955 ***	0.975 ***	0.977 ***	0.180 *	0.393 ***
Gumminess	0.966 ***	0.967 ***	0.955 ***	–0.782 ***	–0.579 ***
Cohesiveness	–0.012	0.180 *	0.792 ***	–0.813 ***	–0.765 ***
Springiness	–0.306 ***	–0.301 **	0.106	–0.702 ***	–0.600 ***

Statistically significant at * *p* < 0.05, ** *p* < 0.01, *** *p* < 0.001.

**Table 3:** The effect of carrot callus cell concentration on the mechanical properties of 0.4% and 1.0% κ-carrageenan–konjac gum–milk hydrogels.

Gel Formulation	Hardness (N)	Cohesiveness	Springiness	Gumminess
0DC-0.4gel	5.94 ± 0.93 ^a^	0.52 ± 0.12 ^a^	0.83 ± 0.07 ^a^	3.05 ± 0.53 ^a^
20DC-0.4gel	6.36 ± 0.90 ^b^	0.41 ± 0.09 ^b^	0.75 ± 0.06 ^b^	2.64 ± 0.68 ^b^
60DC-0.4gel	6.36 ± 0.67 ^b^	0.22 ± 0.04 ^c^	0.66 ± 0.08 ^c^	1.44 ± 0.36 ^c^
0DC-1.0gel	18.00 ± 1.97 ^d^	0.52 ± 0.06 ^ad^	0.78 ± 0.07 ^d^	9.31 ± 1.02 ^d^
20DC-1.0gel	18.56 ± 1.71 ^d^	0.44 ± 0.03 ^be^	0.71 ± 0.06 ^be^	8.35 ± 0.83 ^e^
60DC-1.0gel	19.80 ± 1.84 ^f^	0.38 ± 0.04 ^f^	0.67 ± 0.03 ^cf^	7.58 ± 1.14 ^f^

The data are presented as the mean ± S.D., *n* = 30–55. Different lowercase letters indicate significant differences (*p* < 0.05) between the means.

**Table 4:** Strain sweep for κ-carrageenan–konjac gum–milk hydrogels in the amplitude sweep test (1 Hz. 20 °C).

Parameters	0DC-0.4gel	20DC-0.4gel	60DC-0.4gel	0DC-1.0gel	20DC-1.0gel	60DC-1.0gel
G’_LVE_ (kPa)	2.92 ± 0.07 ^a^	4.70 ± 0.18 ^b^	8.47 ± 0.14 ^c^	11.3 ± 0.6 ^d^	13.8 ± 0.7 ^e^	22.1 ± 0.2 ^f^
G”_LVE_ (kPa)	0.28 ± 0.17 ^a^	0.73 ± 0.03 ^b^	1.60 ± 0.05 ^c^	1.1 ± 0.2 ^d^	2.0 ± 0.3 ^e^	4.2 ± 0.6 ^f^
Tan [δ]_LVE_	0.10 ± 0.01 ^a^	0.16 ± 0.01 ^b^	0.19 ± 0.01 ^c^	0.10 ± 0.03 ^a^	0.15 ± 0.03 ^b^	0.19 ± 0.04 ^c^
Tan [δ]_AF_	0.70 ± 0.25 ^a^	0.53 ± 0.07 ^a^	0.52 ± 0.05 ^a^	0.64 ± 0.11 ^a^	0.69 ± 0.06 ^a^	0.61 ± 0.01 ^a^
τFr (Pa)	57.3 ± 18.7 ^a^	72.3 ± 11.5 ^ab^	98.3 ± 29.4 ^b^	102.9 ± 11.0 ^b^	102.9 ± 11.0 ^b^	175.0 ± 34.7 ^c^
γFr (%)	4.35 ± 0.97 ^a^	3.98 ± 0.32 ^ab^	3.22 ± 0.67 ^b^	1.36 ± 0.57 ^cd^	0.91 ± 0.01 ^c^	1.69 ± 0.26 ^d^

The data are presented as the mean ± S.D., *n* = 6. Different lowercase letters indicate significant differences (*p* < 0.05) between the means. G’_LVE_—the storage modulus, G”_LVE_—the loss modulus, Tan [δ]_LVE_—the loss tangent, Tan [δ]_AF_—the slope of the loss tangent after flow point, τFr—the fracture stress, γFr—the fracture strain.

**Table 5:** The effect of carrot callus cell concentration on the textural qualities of 0.4% and 1.0% κ-carrageenan–konjac gum–milk hydrogels.

Gel Formulation	Hardness	Springiness	Adhesiveness	Swallowability	Juiciness
0DC-0.4gel	10.2 ± 10.4 ^a^	29.1 ± 27.6 ^a^	12.4 ± 13.2 ^a^	88.7 ± 17.9 ^a^	54.3 ± 29.5 ^a^
20DC-0.4gel	16.5 ± 14.8 ^a^	28.9 ± 21.7 ^a^	14.5 ± 13.6 ^a^	85.4 ± 15.5 ^a^	48.5 ± 23.4 ^a^
60DC-0.4gel	28.5 ± 18.4 ^b^	26.5 ± 24.2 ^a^	21.8 ± 21.7 ^b^	76.9 ± 21.5 ^ab^	54.4 ± 23.7 ^a^
0DC-1.0gel	56.5 ± 18.7 ^c^	60.5 ± 25.3 ^b^	15.8 ± 20.1 ^a^	71.6 ± 26.1 ^bc^	33.6 ± 22.5 ^b^
20DC-1.0gel	49.3 ± 22.5 ^c^	50.1 ± 25.6 ^b^	18.0 ± 20.2 ^ab^	72.5 ± 24.6 ^c^	36.7 ± 20.0 ^b^
60DC-1.0gel	52.0 ± 26.5 ^c^	51.0 ± 28.9 ^b^	22.1 ± 21.9 ^b^	65.3 ± 29.9 ^c^	43.5 ± 25.9 ^ab^

The data are presented as the mean ± S.D. ^a, b, c^—*p* < 0.05 between different letters (*n* = 31).

**Table 6:** Correlations (r) between volunteers’ evaluations of textural qualities and the mechanical properties of κ-carrageenan–konjac gum–milk hydrogels.

	Sensory Attributes				
	Hardness	Springiness	Adhesiveness	Swallowability	Juiciness
Hardness (N)	0.652 ***	0.441 ***	0.075	−0.295 ***	−0.276 ***
Cohesiveness	0.050	0.205 **	−0.147 *	0.065	−0.144
Springiness	−0.278	−0.022	−0.183 *	0.216 **	0.003
Gumminess	0.614 ***	0.459 ***	0.024	−0.245 ***	−0.300 ***

*, **, and ***—*p* < 0.05, *p* < 0.01, and *p* < 0.001, respectively.

**Table 7:** Correlations between volunteers’ evaluations of textural qualities of κ-carrageenan–konjac gum–milk hydrogels.

	Graininess	Hardness	Springiness	Adhesiveness	Swallowability	Juiciness
Graininess	-	0.134	0.105	0.207 **	−0.126	0.317 ***
Hardness		-	0.374 ***	0.141	−0.337 ***	−0.128
Springiness			-	0.248 ***	−0.344 ***	−0.109
Adhesiveness				-	−0.316 ***	0.050
Swallowability					-	−0.056
Juiciness						-

** and ***—*p* < 0.01 and *p* < 0.001, respectively.

**Table 8:** The effect of carrot callus cell concentration on the EMG parameters for chewing of 0.4% and 1.0% κ-carrageenan–konjac gum–milk hydrogels.

Gel Formulation	Chewing Time (s)	Number of Chews	M. Masseter (mV × ms)	M. Temporalis (mV × ms)	mm. Suprahyoidei (mV × ms)
0DC-0.4gel	15 ± 8 ^a^	18 ± 9 ^a^	150 ± 94 ^a^	180 ± 140 ^a^	295 ± 193 ^a^
20DC-0.4gel	16 ± 8 ^a^	20 ± 9 ^a^	200 ± 13 ^b^	239 ± 208 ^b^	314 ± 179 ^a^
60DC-0.4gel	17 ± 9 ^a^	20 ± 12 ^a^	181 ± 96 ^b^	275 ± 192 ^c^	340 ± 207 ^a^
0DC-1.0gel	21 ± 10 ^b^	29 ± 15 ^b^	217 ± 106 ^c^	290 ± 166 ^c^	398 ± 178 ^b^
20DC-1.0gel	22 ± 11 ^b^	31 ± 17 ^b^	244 ± 155 ^c^	332 ± 257 ^c^	443 ± 228 ^b^
60DC-1.0gel	22 ± 10 ^b^	31 ± 16 ^b^	280 ± 177 ^d^	411 ± 323 ^d^	457 ± 234 ^c^

The data are presented as the mean ± S.D. ^a, b, c, d^—*p* < 0.05 between different letters (*n* = 31).

**Table 9:** The amplitude and activity of suprahyoid muscles during chewing of 0.4% and 1.0% κ-carrageenan–konjac gum–milk hydrogels by volunteers with normal (*n* = 17) and elevated (*n* = 12) body mass index (BMI).

Gel Formulation	Mean Amplitude (mV)		Activity (mV × ms)	
	Normal BMI	High BMI	Normal BMI	High BMI
0DC-0.4gel	20.3 ± 5.8	16.0 ± 3.5 *	319 ± 202	263 ± 186
20DC-0.4gel	21.4 ± 9.8	16.4 ± 10.5	349 ± 196	252 ± 125
60DC-0.4gel	22.2 ± 5.0	17.0 ± 5.0 *	369 ± 224	281 ± 168
0DC-1.0gel	21.4 ± 4.8	17.2 ± 4.4 *	428 ± 170	333 ± 166
20DC-1.0gel	21.4 ± 4.5	17.4 ± 4.2 *	482 ± 226	372 ± 193
60DC-1.0gel	23.6 ± 5.3	16.8 ± 5.4 *	522 ± 204	339 ± 192 *

The data are presented as the mean ± S.D. *—*p* < 0.05 vs. normal BMI.

## Data Availability

The data that support the findings of this manuscript are available from the corresponding author upon reasonable request.
